# Glycaemic trends, trajectories and control during TB treatment and its association with TB treatment outcome among patients with TB–diabetes multimorbidity

**DOI:** 10.12669/pjms.42.9.19455

**Published:** 2026-09

**Authors:** Maham Zahid, Mahmoud Danaee, Saima Aleem, Saima Afaq

**Affiliations:** 1 Maham Zahid, Institute of Public Health & Social Sciences, Khyber Medical University, Peshawar, Pakistan; 2 Mahmoud Danaee, Department of Social Preventive Medicine, Faculty of Medicine, University of Malaya, Kuala Lumpur, Malaysia; 3 Saima Aleem, Office of Research Innovation and Commercialization, Khyber Medical University, Peshawar, Pakistan; 4 Saima Afaq, Department of Health Sciences, University of York, UK

**Keywords:** Diabetes Mellitus, Glycaemic control, Multimorbidity, Tuberculosis, TB treatment outcome

## Abstract

**Objectives:**

This study aimed to examine longitudinal glycaemic patterns, including trends, trajectories, and control during TB treatment, and their association with treatment outcomes in patients with Tuberculosis (TB) diabetes multimorbidity.

**Methodology:**

Analytical cross-sectional study was conducted, involving secondary analysis of an implementation study conducted at 13 Tuberculosis clinics in KPK and Punjab from June 2022 to June 2025. Random blood glucose (RBG) and glycated haemoglobin were measured at baseline, three months, and six months. Glycaemic trends, trajectories, and control status were reviewed, TB treatment outcomes were assessed at six months and associations were assessed using complete case and imputed datasets.

**Results:**

Among 251 participants, the mean age was 52.0±12.7 years and 60.6% (152/251) were males. Overall, 74.5% (187/251) achieved successful while 64 (25.5%) experienced unsuccessful TB treatment outcomes. TB treatment outcomes showed no significant association with RBG and HbA1c trends or trajectories in the complete case analysis (n=202) However, imputed dataset showed that the occurrence of unsuccessful outcome was higher as compared to successful outcome among those with uncontrolled RBG (96.9% vs 74.3%, p<0.001) and uncontrolled HbA1c (96.6% vs 67.9%, p<0.001), respectively. Binary logistic regression showed higher odds of experiencing unsuccessful outcome as compared to successful outcome due to uncontrolled RBG (OR=6.9; 95%CI: 1.8–27.7) and HbA1c (OR=9.0; 95%CI: 2.3–36.0).

**Conclusion:**

Longitudinal glycaemic patterns showed that most patients with TB–diabetes multimorbidity had uncontrolled glycaemia during treatment, which may affect TB outcomes. Larger studies are needed to better understand these relationships.

## INTRODUCTION

Tuberculosis (TB) and diabetes mellitus are two major global health challenges, particularly in low- and middle-income countries. The coexistence of these conditions presents unique clinical and public health challenges, as diabetes has been associated with increased susceptibility to TB delayed treatment response and poorer clinical outcomes.^1^ WHO report shows that the prevalence of TB-diabetes multimorbidity ranges from 18-35% and the trend is increasing worldwide specially in high TB-burden countries which also have high burden of non-communicable diseases like diabetes.^2^,^3^

The biological link between diabetes and TB is well established, it is evident that a bidirectional relationship exists where host immune response to TB infection can cause hyperglycaemia while hyperglycaemia can increase the susceptibility and severity of TB disease.^4^ Literature also supports the fact that poor glycaemic control can significantly compromise the TB treatment outcomes, which can double the risk of TB treatment failure (OR=1.7), hinders sputum conversion at 3-months, doubles the risk of mortality (OR=1.8), increase risk of disease relapse and development of drug resistant TB among patients suffering from this multimorbidity.^5^-^7^

Another aspect is to explore the longitudinal pattern of glycaemic trends, trajectories and control during the TB treatment period starting from treatment initiation to treatment completion. This requires assessment of blood glucose levels at multiple time points during the TB treatment period.^8^ Some recent studies from India and China have demonstrated dynamic glycaemic trajectories during TB treatment and their potential association with treatment outcomes,^9^ however evidence from Pakistan remains limited. Previous studies have often relied on single-point measurements of blood glucose, which may not adequately capture the dynamic nature of glycaemic control during the course of TB treatment. In contrast, longitudinal patterns—such as trends and trajectories of glucose levels—may provide more meaningful insights into how glycaemic status evolves and impacts treatment response.^10^

Understanding these relationships is particularly important in settings where both TB and diabetes are highly prevalent and healthcare systems are often fragmented. This study includes secondary analysis of data from POTENTIAL project,^11^ in which optimized TB-diabetes care package was introduced in selected TB clinics of KPK and Punjab provinces of Pakistan. This package comprised of glucose monitoring after every 3 months; counselling on life style modification, general TB awareness, treatment adherence for diabetes and TB; and prescription of oral anti-diabetics. For glucose monitoring, the blood glucose levels in terms of random blood glucose and glycated haemoglobin were captured at multiple time points during TB treatment period. The availability of repeated glycaemic measurements during the treatment period provides a unique opportunity to examine dynamic changes in glycaemic status and their potential clinical implications in a real-world setting. Integrated approaches to care require robust evidence on whether improving glycaemic control can meaningfully influence TB outcomes.^11^

In the context of integrated TB–diabetes care in Pakistan, this study examined longitudinal patterns of glycaemic control among patients with TB–diabetes multimorbidity and their relationship with TB treatment outcomes. The aim was to assess glycaemic control using mean levels, temporal trends and trajectories to capture different dimensions of glycaemic status over the course of TB treatment; and to explore their relationship with TB treatment outcomes.

## METHODOLOGY

This analytical cross-sectional study involved a secondary analysis of data from the prospective POTENTIAL cohort of patients with TB–diabetes multimorbidity enrolled in an implementation science study conducted in Pakistan from January 2024 to June 2025.^11^ Data collected during routine study follow-up were analyzed to examine longitudinal patterns of glycaemic control and their association with TB treatment outcomes. The analysis was exploratory in nature and was limited to the variables and measurements available in the parent cohort; consequently, the present study was subject to the timing, completeness, and scope of data collection of the parent study.

### Ethical approval:

Secondary data analysis did not require addition consent from study participants as the consent in original research clearly asked for participant’s permission to allow use of collected data for other research purposes. The research question of this study was different from original research, and is a part of PhD research work done by lead author. The PhD proposal was reviewed and approved by the Ethics Review Committee (ERC) of Institute of Public Health & Social Sciences at Khyber Medical University Peshawar (Ref No. KMU/IPHSS/Ethics/2024/DH/0194; Dated: March 29, 2024).

### Inclusion criteria:

- All consecutive adult participants (18 years or above),
- Diagnosed with drug-sensitive pulmonary TB within past four weeks; either known or newly diagnosed diabetes,
- Registered for TB treatment at selected 13 TB clinics across Khyber Pakhtunkhwa and Punjab province were included in the study.

### Exclusion criteria:

- Participants diagnosed with extra-pulmonary TB,
- Drug-resistant TB,
- History of chronic illnesses including cancer or autoimmune diseases and those who refused to participant in the study were excluded.
- Participants were followed over the standard six-month TB treatment period, with assessments conducted at baseline, three months, and six months. Diabetes status was determined based on prior diagnosis or HbA1c ≥6.5% at baseline.

### Blood glucose measurements:

Blood glucose measurements were done for all study participants. RBG was measured at baseline, three month and six month using glucometers (AccuCheck, Roche) provided to each TB clinic. HbA1c levels were assessed at baseline and six months. At each time point, a 5-mL venous blood sample was collected by a trained TB clinic phlebotomist and transported to the clinic’s in-house laboratory or, where unavailable, to the nearest public-sector laboratory for analysis. HbA1c was measured in whole blood using ion-exchange high-performance liquid chromatography (HPLC) on the Bio-Rad D-10 Hemoglobin Testing System.

RBG and HbA1c measurements were classified using trajectories, trends and control-based approaches. Trajectories, defined as patterns of glycaemic status across time points; trends, reflected direction of change over time; and control status, defined as a composite measure of glycaemic stability and improvement (supplementary Table-SI and Table-SII). RBG was categorized into eight trajectories (High–High–High, High–High–Low, High–Low–High, High–Low–Low, Low–High–High, Low–High–Low, Low–Low–High, Low–Low–Low) where cut-off of ≤180mg/dl and >180mg/dl were used to label low and high, respectively.^12^ Given the limited number of repeated measurements, trajectories were defined using rule-based classification rather than model-based approaches. RBG was categorized in four trends: normal (all readings ≤180 mg/dl), transient (initial >180 mg/dl with subsequent decline to ≤180 mg/dl), erratic (no consistent pattern), and consistently high (all readings >180 mg/dl). On the basis of trends and trajectories, RBG was dichotomized as controlled or uncontrolled (schema given in supplementary Fig.S1).

HbA1cwas categorized into four trajectories (High–High, High–Low, Low–Low, Low–High) where values of ≥8% and <8% was considered indicative of high/low labels, respectively, where ADA defines 8% HbA1c as less-stringent clinically meaningful threshold.^13^ Similarly, it was categorised into three trends: increasing (≥1% rise), no change (<1% change), and decreasing (≥1% reduction), where change of 1% point has been consistently associated with clinically meaningful differences in diabetes-related complications and mortality.^14^ On the basis of trends and trajectories, HbA1c was dichotomized as controlled or uncontrolled (schema given in supplementary Fig.S2)

### TB Outcome measurement:

TB treatment outcomes at six months were classified according to WHO standard definitions including cured, treatment completed, default, treatment failure and death.^15^ TB treatment outcome was dichotomised as successful outcome (cure or treatment completion) and unsuccessful outcome (treatment failure, loss to follow-up, or death).

### Statistical analysis:

Data analysis was performed using IBM SPSS (version 30.0) software. Descriptive statistics were used to summarize baseline characteristics and outcomes. Frequency and percentages were used to present glycaemic trends, trajectories and control, while mean and standard deviations were reported for RBG and HbA1c readings. Overall, there was 19.5% missing data identified in the study, where at 3-months RBG measurements were missing due to deaths (n=5) and lost-to-follow-up (n=23), while at 6-months RBG and HbA1c measurements were missing due to deaths (n=7), and lost to follow up (n=42). Missing data was handled using multiple imputation method based on MICE technique,^16^ where the imputation model included all relevant variables associated with missingness and/or the observed outcomes. Ten imputed datasets were generated, with appropriate imputation models specified according to variable type, and estimates were pooled using Rubin’s rules. A sensitivity analysis was performed to compare results of complete-case (n=202) and imputed data set (n=251). Associations between glycaemic measures and TB treatment outcomes were examined using bivariate analyses, applying chi-square/fisher-exact test depending upon expected cell count. Logistic regression was used to calculate unadjusted odds ratios (OR) with 95% confidence intervals. Multivariable analysis could not be performed due to insufficient number of outcome events and violation of multivariable model assumptions. A p-value of ≤0.05 was considered statistically significant.

## RESULTS

### Sociodemographic characteristics:

Data from 251 patients with TB-diabetes multimorbidity was included in secondary analysis. Overall, the mean age was 52.0 ± 12.7 years, and 60.6% of the study population comprised of male gender. Majority of the participants, 204 (81.3%) had a known history of diabetes, while remaining 47 (18.7%) were newly diagnosed. Among those with previous history of diabetes, mean duration of diabetes was 7.0±4.9 years, and 173 (84.8%) were on anti-diabetic drugs. Table-I summarizes baseline demographic and clinical characteristics.

**Table-I:** Distribution of baseline demographic characteristics of participants with TB-diabetes (n=251).

	Characteristics	Frequency (%)
Age	Mean age (years)	52.0±12.7
Gender	Male	152 (60.6%)
	Female	99 (39.4%)
Residence	Urban	82 (32.7%)
	Rural	169 (67.3%)
Socioeconomic status	Low	48 (19.1%)
	Middle	189 (75.3%)
	High	14 (5.6%)
Employment status	Unemployed	144 (57.4%)
	Employed	107 (42.6%)
Smoking status	Current smoker	23 (9.2%)
	Ex-smoker	55 (21.9%)
Nutritional status	Under weight (BMI <18.5)	70 (27.9%)
	Normal or more than normal weight (BMI >18.5)	181 (72.1%)
Diabetes status	Known history of diabetes	204 (81.3%)
	Newly diagnosed diabetes	47 (18.7%)
Severity of TB disease	Mild	55 (21.9%)
	Moderate	157 (62.5%)
	Severe	39 (15.5%)

### Glycaemic Trends, trajectories and control:

A summary of glycaemic trends, trajectories and control for both RBG and HbA1c (complete case vs imputed dataset analyses), shown in Table-II.

**Table-II:** Summary of glycaemic trends, trajectories and control among participants with TB-diabetes multimorbidity during TB treatment period.

		Complete case analysis (n=202) - n(%)	Imputed data set (n=251) - n(%)
***Random Blood Glucose (RBG)***			
Trends	Normal	19 (9.4%)	19 (7.6%)
	Transient	30 (12.4%)	27 (10.8%)
	Erratic	72 (24.2%)	61 (24.2%)
	Consistently high	81 (54.0%)	144 (57.4%)
Trajectories	High–High–High	109 (54.0%)	144 (57.4%)
	High–High–Low	11 (5.4%)	11 (4.4%)
	High–Low–High	21 (10.4%)	23 (9.2%)
	High–Low–Low	19 (9.4%)	19 (7.5%)
	Low–High–High	19 (9.4%)	31 (12.4%)
	Low–High–Low	1 (0.5%)	1 (0.4%)
	Low–Low–High	3 (1.5%)	3 (1.2%)
	Low–Low–Low	19 (9.4%)	19 (7.5%)
Control	Controlled	50 (24.8%)	50 (19.9%)
	Uncontrolled	152 (75.2%)	201 (80.1%)
***Glycated Haemoglobin (HbA1c)***			
Trends	Increasing trend	34 (16.8%)	42 (16.7%)
	No change	58 (28.7%)	71 (28.3%)
	Decreasing trend	110 (54.5%)	138 (55.0%)
Trajectories	High-High	130 (64.4%)	171 (68.1%)
	High-Low	33 (16.3%)	33 (13.1%)
	Low-Low	29 (14.4%)	29 (11.6%)
	Low-High	10 (5.0%)	18 (7.2%)
Control	Controlled	62 (30.7%)	62 (24.7%)
	Uncontrolled	140 (69.3%)	189 (75.3%)

With respect to RBG trends, out of 202 complete cases, majority, 81 (54.0%) exhibited consistently high RBG level, erratic trend was observed in 72 (24.2%) participants, while fewer participants 30 (14.9%) showed transient and 19 (9.4%) had normal trend. In terms of trajectories, over half of participants followed a High–High–High pattern 100 (53.5%) while only 18 (9.4%) followed High-Low-Low pattern. Overall, majority of the participants 152 (75.2%) exhibited uncontrolled RBG throughout TB treatment period.

For HbA1c, more than half of the participants, 102 (54.5%) demonstrated a decreasing trend, while 54 (28.9%) showed no change and 31 (16.6%) had increasing HbA1c trend. The dominant trajectory was High–High among 118 (63.1%) participants, indicating persistently elevated HbA1c levels, while only 33 (17.6%) participants showed High-Low HbA1c pattern. Overall, findings from both complete case and imputed analyses were consistent, indicating a high burden of poor glycaemic control throughout the treatment period. Overall, HbA1c remained uncontrolled in majority of the participants, 62 (30.7%) during TB treatment.

### Association with TB treatment outcomes:

At end of TB treatment period, there were total 72 (28.7%) participants who completed treatment, 115 (45.8%) were cured, 15 (6.0%) had treatment failure, 42 (16.7%) did not complete the treatment and thus were defaulted, while 7 (2.8%) deaths were reported. Combining these into successful and unsuccessful outcome, there were 187 (74.5%) and 64 (25.5%) successful and unsuccessful outcomes, respectively. The missing data was primarily due to deaths and defaulters, both of which were categorized as unsuccessful TB treatment outcome, therefore in the imputed analysis 49 cases were added in this group.

No significant difference was observed in mean RBG and HbA1c at different timepoints with respect to TB treatment outcomes, as shown in Fig.1. Similarly, TB treatment outcomes showed no significant association with RBG and HbA1c trends or trajectories in the complete case analysis (n=202) as given in Table-III. However, in the imputed dataset (n=251) some significant associations were noticed for both RBG and HbA1c trends/trajectories. A higher proportion of participants with unsuccessful outcomes exhibited consistently high RBG trends compared to those with successful outcomes (68.8% vs 53.5%, p=0.029). Similarly, unsuccessful outcomes were more frequently observed among participants with high–high–high (68.8% vs 53.5%, p=0.002) and low–high–high (21.9% vs 9.1%, p=0.002) RBG trajectories. Additionally, a greater proportion of participants with unsuccessful outcomes had uncontrolled RBG as compared to those with successful outcomes (96.9% vs 74.3%, p<0.001).

**Fig.1:**
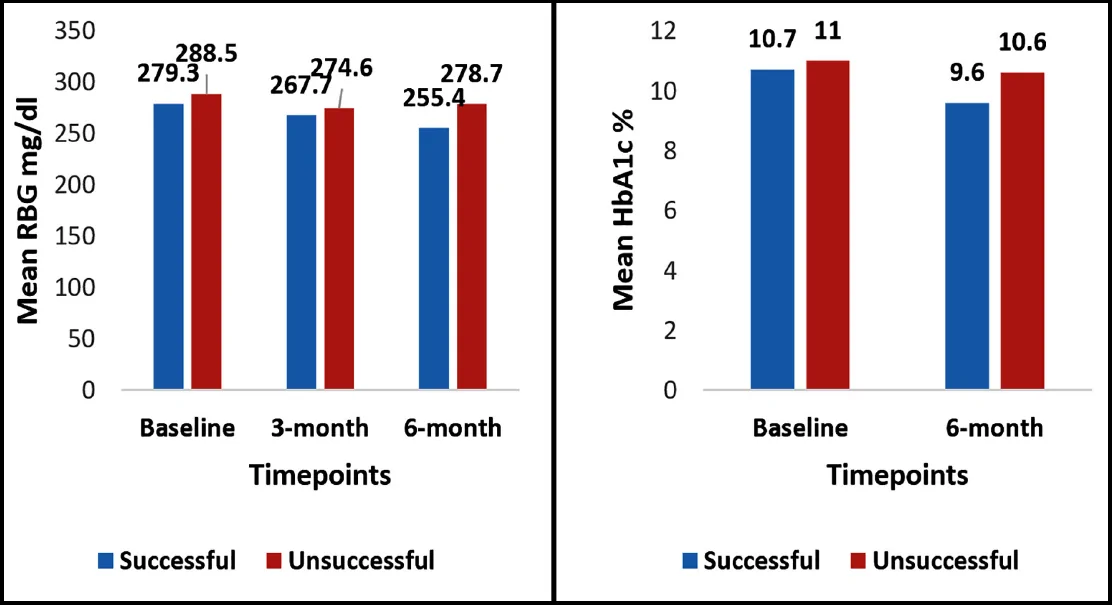
Comparison of mean RBG and mean HbA1c at various timepoints between successful and unsuccessful TB treatment outcome groups.

**Table-III:** Association of TB treatment outcome with glycaemic trends and trajectories

	Complete case analysis		p	Imputed data set		P
	Successful outcome (n=187)	Unsuccessful outcome (n=15)		Successful outcome (n=187)	Unsuccessful outcome (n=64)	
RBG trends						
Normal	18 (9.6%)	1 (6.7%)	0.872	18 (9.6%)	1 (1.6%)	0.029
Transient	24 (12.8%)	1 (6.7%)		24 (12.8%)	3 (4.7%)	
Erratic	45 (24.1%)	5 (33.3%)		45 (24.1%)	16 (25.0%)	
Consistently high	100 (53.5%)	8 (53.3%)		100 (53.5%)	44 (68.8%)	
RBG trajectories						
High-High-High	100 (53.5%)	9 (60.0%)	0.691	100 (53.5%)	44 (68.8%)	0.002
High-Low-High	20 (10.7%)	1 (6.7%)		20 (10.7%)	3 (4.7%)	
High-Low-Low	18 (9.6%)	1 (6.7%)		18 (9.6%)	1 (1.6%)	
High-High-Low	11 (9.1%)	0 (0.0%)		11 (5.9%)	0 (0.0%)	
Low-High-Low	1 (0.5%)	0 (0.0%)		1 (0.5%)	0 (0.0%)	
Low-High-High	17 (9.1%)	2 (13.3%)		17 (9.1%)	14 (21.9%)	
Low-Low-Low	18 (9.6%)	1 (6.7%)		18 (9.6%)	1 (1.6%)	
Low-Low-High	2 (1.1%	1 (6.7%)		2 (1.1%)	1 (1.8%)	
RBG control						
Controlled	48 (25.7%)	2 (13.3%)	0.367	48 (25.7%)	2 (3.1%)	<0.001
Uncontrolled	139 (74.3%)	13 (86.7%)		139 (74.3%)	62 (96.9%)	
HbA1c trends						
Decreasing	102 (54.5%)	8 (53.3%)	0.940	102 (54.5%)	36 (56.3%)	0.939
No change	54 (28.9%)	4 (26.7%)		54 (28.9%)	17 (26.6%)	
Increasing	31 (16.6%)	3 (20.0%)		31 (16.6%)	11 (17.2%)	
HbA1c trajectories						
High-High	118 (63.1%)	12 (80.0%)	0.338	118 (63.1%)	53 (82.8%)	<0.001
High-Low	33 (17.6%)	0 (0.0%)		33 (17.6%)	0 (0.0%)	
Low-High	9 (4.8%)	1 (6.7%)		9 (4.8%)	9 (14.1%)	
Low-Low	27 (14.4%)	2 (13.3%)		27 (14.4%)	2 (3.1%)	
HbA1c control						
Controlled	60 (32.1%)	2 (13.3%)	0.156	60 (32.1%)	2 (3.1%)	<0.001
Uncontrolled	127 (67.9%)	13 (86.7%)		127 (67.9%)	62 (96.6%)	

*Fisher-exact test was applied where the expected cell count was less than 5.

With respect to HbA1c trends, significantly higher rate of unsuccessful outcome was reported among high–high trend as compared to successful outcomes (82.8% vs 63.1%; p<0.001); on the other hand, higher successful outcome rate was observed among low-low trend as compared to unsuccessful outcomes (14.4% vs 3.1%; p<0.001). Additionally, a greater proportion of participants with unsuccessful outcomes had uncontrolled HbA1c as compared to those with successful outcomes (96.6% vs 67.9%, p<0.001).

Binary logistic regression, using imputed data-set (n=251), showed a significant association between uncontrolled RBG and unsuccessful TB treatment outcomes where participants with uncontrolled RBG had higher odds to experience unsuccessful outcomes as compared to those with controlled RBG (OR=6.9; 95% CI: 1.8–27.7). Similarly, odds of unsuccessful TB treatment outcomes were higher among those with uncontrolled HbA1c as compared to those with controlled HbA1c (OR=9.0; 95% CI: 2.3–36.0). The wide confidence intervals show small number of unsuccessful events as compared to successful events, and less previse findings.

## DISCUSSION

This study examined longitudinal glycaemic patterns and their relationship with TB treatment outcomes among patients with TB–diabetes multimorbidity receiving optimized TB-diabetes care. A substantial proportion of participants had poor glycaemic control at treatment initiation, and only modest improvement was observed during the course of TB treatment, with many participants remaining inadequately controlled at treatment completion. Persistent hyperglycaemia and glycaemic instability were more frequently observed among participants with unsuccessful TB treatment outcomes, although these associations did not reach statistical significance in the primary complete-case analysis.

The findings are broadly consistent with previous studies reporting a high proportion of hyperglycaemia among people with TB and diabetes or TB–diabetes multimorbidity at the initiation of TB treatment. Lui Q et al. reported that 24% of participants experienced transient hyperglycaemia and 10% experienced persistent hyperglycaemia during TB treatment, with both patterns associated with increased odds of adverse treatment outcomes compared with consistently normoglycaemic individuals (aOR=4.2, p=0.004).^17^ Similarly, Pardeshi G et al. found that approximately 30% of participants had either transient or persistent hyperglycaemia, with persistent hyperglycaemia significantly associated with unfavourable treatment outcomes (aRR=2.07, p<0.001), whereas transient hyperglycaemia was not significantly associated with outcomes.^9^ Kornfeld H et al., in a secondary analysis of two cohorts, further demonstrated that TB may be accompanied by transient elevations in blood glucose that do not necessarily progress to chronic dysglycaemia, while increasing HbA1c during and after TB treatment may be associated with unfavourable outcomes, particularly among individuals with HbA1c ≥6.5% at diagnosis.^18^

In terms of association between glycaemic control and TB treatment outcomes, recent systematic reviews have reported weak associations between glycaemic control and TB treatment outcomes and considerable heterogeneity across studies.^8^,^19^ It is reported that patients with uncontrolled high glucose can approximately have two times higher risk of TB treatment failure or unsuccessful outcome (RR = 1.91, 95% CI 1.81–3.07, p=0.008), and around three times higher risk of sputum positivity after 3 months of TB treatment (RR = 2.97, 95% CI 1.10–8.07, p = 0.03).^20^ However, a range of risk estimates are reported among different studies which can be due to differences in study populations, definitions of glycaemic control, timing and frequency of measurements, outcome definitions, and adjustment for confounding. In particular, much of the existing literature has focused on comparisons between TB individuals with and without diabetes, while fewer studies have specifically examined longitudinal glycaemic patterns among individuals with established TB–diabetes multimorbidity. The present study therefore adds to a relatively limited body of evidence by examining changes in glycaemic status over the course of TB treatment within this high-risk population.

The clinical relevance of these findings lies in the potential value of incorporating serial glycaemic assessment into integrated TB-diabetes care. Persistent or worsening hyperglycaemia during TB treatment may identify patients who require closer clinical review, including assessment of diabetes treatment, adherence and other factors affecting glycaemic control. Serial HbA1c and blood glucose measurements may also help distinguish transient dysglycaemia associated with acute TB illness from persistent poor glycaemic control requiring ongoing diabetes management. This could provide an opportunity for earlier identification of patients at potentially greater risk and support more individualized management within integrated TB-diabetes services.^21^ However, the findings should not be interpreted as evidence that improving glycaemic control will necessarily improve TB treatment outcomes. Prospective interventional studies are needed to determine whether trajectory-guided management of hyperglycaemia can translate into better TB outcomes before such an approach can be recommended as part of routine TB care.

### Limitations

As a secondary analysis, the study was not specifically designed or powered to investigate the associations, rather this was exploratory in nature. Although the overall cohort included 251 participants, only 15 unsuccessful treatment outcomes were available for the complete-case analysis. This substantially limited statistical power and precision, precluded reliable multivariable adjustment, and resulted in wide confidence intervals in the sensitivity analyses. Consequently, the absence of statistical significance in the primary analysis may partly reflect limited power rather than the absence of a true association. The differences between the complete-case and imputed analyses further warrant cautious interpretation of the findings. Several factors, including age, duration of diabetes, baseline glycaemic status, BMI, smoking, diabetes treatment and TB severity, may influence both glycaemic trajectories and treatment outcomes. However, the small number of unsuccessful outcomes prevented reliable adjustment for multiple potential confounders, and residual confounding cannot therefore be excluded. Furthermore, because this was an observational secondary analysis, causal relationships between glycaemic trajectories and TB treatment outcomes cannot be established, and limitations related to the availability and timing of measurements may also have influenced the observed associations.

### Strengths

The longitudinal design enabled assessment of patterns of glycaemic control during TB treatment rather than relying exclusively on a single baseline measurement. Missing data were addressed using multiple imputation, and sensitivity analyses were conducted to examine the robustness of the findings. In addition, this study contributes evidence from Pakistan, where longitudinal evidence on glycaemic trajectories among people with TB–diabetes multimorbidity remains limited. The findings therefore provide a basis for further investigation and support the need to consider glycaemic control as a dynamic component of TB-diabetes management rather than a static characteristic assessed only at treatment initiation.

## CONCLUSION

Poor glycaemic control remained common throughout TB treatment, with only modest improvement over time. While the primary analysis found no statistically significant association between glycaemic patterns and TB treatment outcomes, sensitivity analysis suggested a potential relationship between elevated glycaemia and unfavorable outcomes. These findings support the potential value of longitudinal glycaemic monitoring within integrated TB-diabetes care, but larger prospective studies are needed to confirm these associations and determine their clinical significance.

### Recommendations

Future research should build on these findings through larger, adequately powered prospective studies with predefined glycaemic trajectories, standardized schedules for serial HbA1c and glucose assessment, and sufficient numbers of unsuccessful treatment events to permit robust multivariable adjustment. Such studies should also collect detailed information on potential confounders, including diabetes duration and treatment, adherence, nutritional status, TB severity and other clinical factors. Further research could also explore whether combining longitudinal glycaemic measures with other clinical or biological markers improves prediction of unfavorable TB outcomes. This evidence could ultimately inform integrated, patient-centred TB-diabetes care models and clarify whether trajectory-guided glycaemic management has a role in routine TB services.

### Author’s Contributions:

**MZ:** Conceptualization, study design, data analysis, manuscript drafting, research integrity

**MD:** Data analysis, and final manuscript review.

**SAL:** Literature search, manuscript revision.

**SA:** Conceptualization, supervision and accountable for the accuracy of the study.

All authors have approved the final version of the manuscript.
